# The Safety and Efficacy of the Repeated PRRT with [^90^Y]Y/[^177^Lu]Lu-DOTATATE in Patients with NET

**DOI:** 10.1155/2021/6615511

**Published:** 2021-01-23

**Authors:** Anna Zemczak, Paweł Gut, Dariusz Pawlak, Maciej Kołodziej, Leszek Królicki, Beata Kos-Kudła, Marek Ruchała, Grzegorz Kamiński, Jolanta Kunikowska

**Affiliations:** ^1^Department of Endocrinology and Neuroendocrine Tumours, Department of Pathophysiology and Endocrinology, Medical University of Silesia, Katowice, Poland; ^2^Department of Endocrinology, Metabolism and Internal Diseases, Poznan University of Medical Sciences, Poznan, Poland; ^3^Radioisotope Centre POLATOM, National Centre for Nuclear Research, Otwock, Poland; ^4^Department of Endocrinology and Radioisotope Therapy, Military Institute of Medicine, Warsaw, Poland; ^5^Nuclear Medicine Department, Medical University of Warsaw, Warsaw, Poland

## Abstract

**Purpose:**

The peptide receptor radionuclide therapy (PRRT) is a treatment option for patients with disseminated, inoperable G1 and G2 neuroendocrine tumours (NETs). The study aims to evaluate the safety, efficacy, and progression-free survival (PFS) of patients after retreatment (R-PRRT) and re-retreatment (RR-PRRT) with tandem isotopes [^90^Y]Y/[^177^Lu]Lu-DOTATATE. *Material and Methods*. Out of 99 treated patients with G1 and G2 NETs, 26 were included in the study and treated with the repeated PRRT (with 5 undergoing the re-repeated PRRT treatment) after an initial positive response to four PRRT cycles and later progression of the disease. [^68^Ga]Ga-DOTATATE PET/CT and CT/MRI procedures were performed before and after the treatment. Patients were treated with [^90^Y]Y/[^177^Lu]Lu-DOTATATE (1 : 1) with mixed amino acid infusion for kidney protection. Toxicity was evaluated using the CTCAE 3.0 criteria.

**Results:**

The median follow-up was 88 months (the range: 42–164). The median cumulative administered activity was 22.2 GBq (the range: 17.8–30.7 GBq). Myelodysplastic syndrome occurred in one patient (3.8%), and grade 4 renal toxicity was also detected in one patient (3.8%). No other cases of grade 3 or 4 bone marrow and renal toxicity were observed. The median PFS rate was 31 months after the PRRT and 23 months following the R-PRRT. The OS rate from the diagnosis (OS-d) was 109 months and from the start of the PRRT (OS-t)-92.4 months. During the restaging, 3–6 months after the PRRT, PR, SD, and PD were observed in 19.2%, 80.8%, and 0% of the patients, respectively. After the R-PRRT, PR, SD, and PD were observed in 50%, 42.3%, and 7.7% of the patients, respectively.

**Conclusions:**

The repeated therapy with [^90^Y]Y/[^177^Lu]Lu-DOTATATE is safe and effective for patients with disseminated, inoperable G1 and G2 neuroendocrine tumours.

## 1. Introduction

The presence of somatostatin receptors on the surface of cells of neuroendocrine tumours (NETs), mainly G1 and G2 ones, has been used both in the diagnostic process—in somatostatin receptor imaging (SRI)—and in therapy—in radioisotope-labelled somatostatin analogues with high affinity for somatostatin receptor subtype 2 (sstr2), the most common of SSTRs [1]. The peptide receptor radionuclide therapy (PRRT) has been used to treat NETs for more than 25 years.

Initially, Auger electron emitter ^111^In was used to treat neuroendocrine tumours. Unfortunately, due to the physical properties of ^111^In, the obtained PRRT results were limited in scope and observed only in 8% of patients. The observed rates of grade 3 or 4 bone marrow and renal toxicity were low and transient [2]. Myelodysplastic syndrome (MDS) or leukaemia was observed in 6% of the patients who received doses higher than 100 GBq [3].

*β* emitters were introduced next: first, ^90^Y—which emits high energy radiation and has a tissue penetration range of up to 10 mm—and, later on, ^177^Lu—which emits beta radiation with lower energy and has the tissue penetration range of up to 4 mm. Studies of efficacy of [^90^Y]Y-DOTATOC and [^177^Lu]Lu-DOTATATE have shown that a positive response to treatment was recorded in 15–35% of the analyzed patients [4–8].

Initially, nephrotoxicity was the main side effect of such treatments. However, once application of amino acid infusion during the PRRT was introduced to the clinical practice, the level of nephrotoxicity was reduced, becoming more common after application of ^90^Y, or sequential use of ^90^Y and ^177^Lu, than in the case of any treatment conducted with ^177^Lu alone (2.8% *vs.* 1.3 *vs.* 0%, respectively) [9]. Other PRRT-focused studies have confirmed greater nephrotoxicity in patients treated with ^90^Y than ^177^Lu [6–8, 10].

Mild levels of haematological toxicity (grade 1 or 2) were recorded most commonly [6, 9]. Grade 3 or 4 bone marrow toxicity developed in 9.5% of cases and occurred more frequently after application of ^90^Y than ^177^Lu. Myelodysplastic syndrome occurred in 2.35% of the patients, and leukaemia, in 1.1% [9].

The absorbed doses—23 Gy to the kidneys and 2 Gy to the bone marrow—were established on the basis of dosimetry data [11–14].

The results of the first randomized, phase 3 trial, NETTER-1, published in 2017 by Strosberg et al., proved that the survival parameters in the patients with midgut NET treated with [^177^Lu]Lu-DOTATE and 30 mg octreotide were improved compared with the patients who were treated with only 60 mg octreotide. The researchers reported that transient grade 3 or 4 neutropenia, thrombocytopenia, and lymphopenia occurred in 1%, 2%, and 9% of the patients, respectively. No nephrotoxicity was observed during the 14-month follow-up period [15].

The variety of sizes of metastatic tumours occurring in patients supports the logic of simultaneous use of [^90^Y]Y/[^177^Lu]Lu-DOTATATE. Previously published data have proven that this kind of combined treatment has greater efficacy than application of [^90^Y]Y-DOTATATE alone, while the side effects of both methods remain comparable. A Polish multicenter trial has confirmed that tandem [^90^Y]Y/[^177^Lu]Lu-DOTATATE therapy increases the overall survival rate in a more significant way than single-isotope treatment with [^90^Y]Y-DOTATATE and [^177^Lu]Lu-DOTATATE. Haematological toxicity rarely occurred in the patients undergoing the treatment. Renal toxicity was observed only in the patients treated with [^90^Y]Y-DOTATATE—grade 3 in 5–12% and grade 4 in 3–8% [16]. A subsequent trial using simultaneously [^90^Y]Y/[^177^Lu]Lu-DOTATATE in patients with disseminated/inoperable G1/G2 NET showed that this tandem therapy is both highly effective and safe, also in terms of its long-term side effects [17, 18].

In some patients, disease progression is still observed despite the positive response to the PRRT.

If disease progression occurs after effective application of the PRRT, repeated cycles of the PRRT might be considered as a further treatment option. However, there probably means an increased risk of occurrence of nephrotoxicity and haematological toxicity, as the cumulative absorbed doses are higher.

Only a few studies concerning possibility of retreatment with the radioisotope therapy could be found in the literature of the subject. However, some of the publications concerning studies involving limited numbers of patients presented only results of reapplication of [^177^Lu]Lu-DOTATATE [19–24], and only one paper analyzed repeated cycles of treatments with [^90^Y]Y-DOTATATE, [^177^Lu]Lu-DOTATATE, and [^90^Y]Y/[^177^Lu]Lu-DOTATATE [25].

The main aim of this study was to evaluate the safety of retreatment (R-PRRT) and re-retreatment (RR-PRRT) with tandem isotopes [^90^Y]Y/[^177^Lu]Lu-DOTATATE. The secondary endpoint was to evaluate their efficacy, as well as progression-free survival (PFS).

## 2. Materials and Methods

### 2.1. Patients

Out of 99 patients with G1 and G2 metastatic NETs, 26 patients were treated with a repeated tandem [^90^Y]Y/[^177^Lu]Lu-DOTATATE therapy (R-PRRT), including 5 patients who underwent re-repeated therapy (RR-PRRT), after the initial favourable response to four PRRT cycles and later progression of the disease.

All the patients met the following inclusion criteria:

- Histological confirmation of G1 or G2 neuroendocrine tumour (NET) and metastatic disease
- Preserved haematological, liver, and renal parameters: haemoglobin ≥10 g/dL, white blood cell (WBC) count ≥3 × 10^9^/L, platelet count ≥90 × 10^9^/L, bilirubin ≤1.5 × upper limit of normal (ULN), ALT < 2.5 × ULN, and estimated creatinine clearance (CrCl) > 40 mL/min
- Positive somatostatin receptor imaging (SRI)—PET/CT using [^68^Ga]Ga-DOTATATE with uptake equal to or higher than that in the liver
- Karnofsky index ≥70 and ECOG performance status ≤2
- Age >18 years
- Life expectancy >3 months
- No pregnancy or lactation
- Objective response (CR, PR) and stable disease (SD) after the PRRT and R-PRRT in case of progression in the CT or MRI examination and SRI.

The exclusion criterion was patients with mismatch lesion: positive in [^18^F]FDG and negative in [^68^Ga]Ga-DOTATATE PET/CT.

The study was conducted in accordance with the principles of the Declaration of Helsinki and Good Clinical Practice guidelines. This multi-institution study was approved by the Ethical Committees of the Medical University of Warsaw, the Military Institute of Medicine of Warsaw, and the University of Medical Sciences in Poznan. All participating patients gave written informed consent.

### 2.2. Study Treatment and Radiopeptide Administration

Tandem [^90^Y]Y/[^177^Lu]Lu-DOTATATE therapy, consisting of a mix of 50% radioactivity of [^90^Y]Y-DOTATATE (1.48–1.85 GBq) and 50% radioactivity of [^177^Lu]Lu-DOTATATE (1.48–1.85 GBq), with a 1 : 1 treatment ratio, was prepared with previously described methods, using ^90^Y and ^177^Lu (ItraPol and LutaPol; POLATOM, Poland) [17, 26, 27].

All of 26 patients participating in our study received earlier 4 cycles of 2.96–3.7 GBq of tandem [^90^Y]Y/[^177^Lu]Lu-DOTATATE therapy with amino acid infusion for nephroprotection, with treatment procedure as previously described [27, 28]. The intervals between cycles were 6‐ to 12-weeks long, with their exact length dependant on the patients' clinical condition, results of laboratory tests, examinations, and radiopharmaceutical availability. The patients qualified for the repeated radioisotope treatment were the patients for whom objective response or stable disease was observed at least one year after the initial PRRT and who later registered progression of the disease. They were given 2 additional cycles of 2.96–3.7 GBq of the radioisotope treatment. Five patients who responded well to the previous treatment (PRRT and R-PRRT) have received two more cycles of the radioisotope treatment after subsequent progression. The inclusion criteria for the PRRT were applied also to the R-PRRT and RR-PRRT.

The PRRT was provided to patients receiving long-acting somatostatin analogues 4–5 weeks after completing the therapy with octreotide (Sandostatin LAR; Novartis) and 5–7 weeks after completing the therapy with lanreotide (Somatuline Autogel; Ipsen). This treatment was continued during the PRRT, R-PRRT, and RR-PRRT. The PRRT was performed only if more than 3 months have passed since the end of chemotherapy treatments.

### 2.3. Post-Therapy Imaging

Post-therapy imaging was performed 24 hours after the therapy, allowing for monitoring of biodistribution during the treatment. The acquisition was made with an energy window of ±10% centred on ^177^Lu photopeaks (208 keV), as previously described [17, 26, 27].

### 2.4. Assessment of the Treatment Results and the Clinical Benefits

The main aim of the study was to evaluate toxicity of repeated cycles of the PRRT. The secondary endpoint was to evaluate the therapy's efficacy and its rate of progression-free survival (PFS) defined as the time counted from the start of the radioisotope treatment, as well as retreatment and re-retreatment, to discovering the first evidence of progression by imaging criteria (CT/MRI and/or SRI) or the patient's death from any cause.

Additionally, we investigated also the overall survival parameters. The overall survival rate from the diagnosis (OS-d) was calculated from the first diagnosis of the tumour to death from any cause. The OS rate from the start of the treatment (OS-t) was defined as the time from the first cycle of [^90^Y]Y/[^177^Lu]Lu-DOTATATE treatment to death from any cause.

The response to treatment could be defined as one of the following: objective response (complete response (CR) and partial response (PR)), stable disease (SD), and progressive disease (PD), in accordance with the Response Evaluation Criteria in Solid Tumours 1.1 (RECIST 1.1) for radiological evaluation and SRI. The disease control rate (DCR) was defined as the proportion of patients who achieved OR and SD.

All the patients underwent staging and restaging by contrast-enhanced CT or MRI and [^68^Ga]Ga-DOTATATE PET/CT.

The assessment of response to treatment after completing the PRRT, R-PRRT, and RR-PRRT was done after 3–6 months, after first 12 months, and every 12 months thereafter, with the help of diagnostic imaging and blood markers.

Blood tests for full blood cell count and kidney and liver function parameters were repeated every 7–21 days after each therapy cycle, as well as 3, 6, and 12 months after completing the therapy, and every 12 months thereafter. The toxicity of the PRRT was evaluated using the Common Terminology Criteria for Adverse Events v3.0 (CTCAE).

Kidney function was assessed using the modification of diet in renal disease formula.

### 2.5. Statistical Methods

Mean values and standard deviations, as well as medians and quartiles or frequencies, depending on the parameter distribution, were used to summarize patients' characteristics. The calculations were done using Excel (2007 version, Microsoft).

The OS and PFS rates were calculated using the Kaplan–Meier estimator and compared using the log-rank test. The calculations were done using GraphPad PRISM 5 (GraphPad Software Inc).

## 3. Results

### 3.1. Patients' Characteristics

Before the first cycle of tandem [^90^Y]Y/[^177^Lu]Lu-DOTATATE therapy, 25 patients underwent surgery, 4 patients received chemotherapy, and 1 patient was treated with sunitinib. Long-acting somatostatin analogues were also used before in 19 patients (13 with octreotide and 6 with lanreotide), during and in follow-up of the radioisotope therapy. Only one patient received chemotherapy due to disease progression between the PRRT and R-PRRT. All the patients had positive [^68^Ga]Ga-DOTATATE PET/CT study in all the defined lesions. The baseline characteristics of the patients' data are shown in Table 1.

### 3.2. Toxicity of [^90^Y]Y/[^177^Lu]Lu-DOTATATE Therapy

All the participating patients have demonstrated good tolerability of the PRRT, without any serious or acute adverse events. Nausea and fatigue were the most frequent side effects reported during the treatment. The levels of bone marrow and renal toxicity after application of the PRRT, R-PRRT, and RR-PRRT are presented in Tables 2 and 3.

Among patients who developed bone marrow toxicity after the initial PRRT, grade 1 toxicity was observed in 9 patients (34.6%) and transient grade 2 toxicity was observed in 2 patients (7.7%). After the R-PRRT, grade 1 haematological toxicity was developed in 15 patients (57.7%), while grade 2 toxicity occurred in 4 patients (15.4%). After the RR-PRRT, grade 1 bone marrow toxicity was detected in 3 patients, and grade 2 bone marrow toxicity, in 1 patient. Only one patient (3.8%) developed myelodysplastic syndrome (MDS) after receiving the cumulative administrated dose of 30.7 GBq. The patient was 65 years old during the RR-PRRT and was previously treated with two lines of chemotherapy because of a G2 pancreatic NET with numerous metastases to liver, lymph nodes, and bones. He was the only patient out of all the patients treated with the RR-PRRT who had earlier undergone chemotherapy. No other cases of grade 3 and 4 toxicity were observed after the initial and repeated PRRT.

Grade 1 renal toxicity was developed in 5 patients (19.2%), and in two of those cases, the toxicity was transient after the initial PRRT. At the R-PRRT stage, there was grade 1 renal toxicity observed in 7 patients (26.9%) and grade 2 renal toxicity in 2 patients (7.7%). After the RR-PRRT, grade 1 and grade 2 nephrotoxicity were observed in 1 patient each. Only one (3.8%) 77-year-old patient developed grade 4 renal toxicity, which requires dialysis after receiving the cumulative administered dose of 29.6 GBq. This patient was diagnosed with right ventricular failure due to carcinoid heart disease and had hypertension and a history of acute glomerulonephritis (probably postinfectious). No other cases of grade 3 and 4 toxicity were observed after the initial and repeated PRRT.

No cases of hepatotoxicity of any grade were observed.

### 3.3. Results of Tandem [90Y]Y/[177Lu]Lu-DOTATATE Therapy

The median duration of follow-up, counted from the start of the therapy, was 88 months (the range: 42–164 months). Twelve patients died during the follow-up stage. The median total cumulative administered activity was 22.2 GBq (the range: 17.8–30.7 GBq). Twenty-six patients received the R-PRRT, and five received RR-PRRT.

As the group of patients treated with the RR-PRRT was small, they were not subjected to a detailed analysis, and the results obtained for those patients are not reliable.

**Figure 1:**
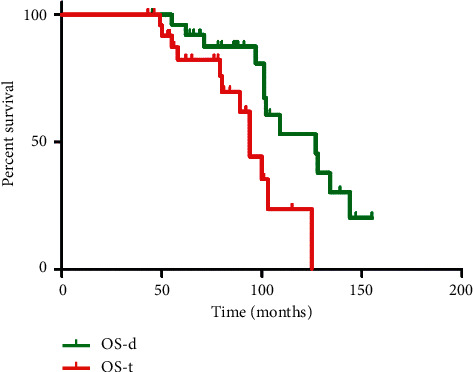
Tandem [^90^Y]Y/[^177^Lu]Lu-DOTATATE therapy: Kaplan–Meier estimators of the overall survival from the diagnosis (OS-d) in relation to the overall survival from the time of therapy (OS-t).

The OS rate from diagnosis (OS-d) was 109 months, and 92.4 months was the rate for the overall survival time from the start of the PRRT (OS-t) (Figure 1).

**Figure 2:**
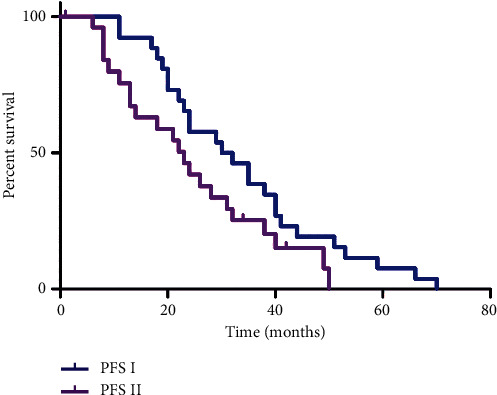
Tandem [^90^Y]Y/[^177^Lu]Lu-DOTATATE therapy: Kaplan–Meier estimators of the progression-free survival after the initial PRRT (PFS I) in relation to the progression-free survival after the repeated PRRT (PFS II).

The median PFS after the initial PRRT was 31 months, which was statistically longer than the PFS period after the R-PRRT, which amounted to 23 months (*p*=0.048) (Figure 2).

**Figure 3:**
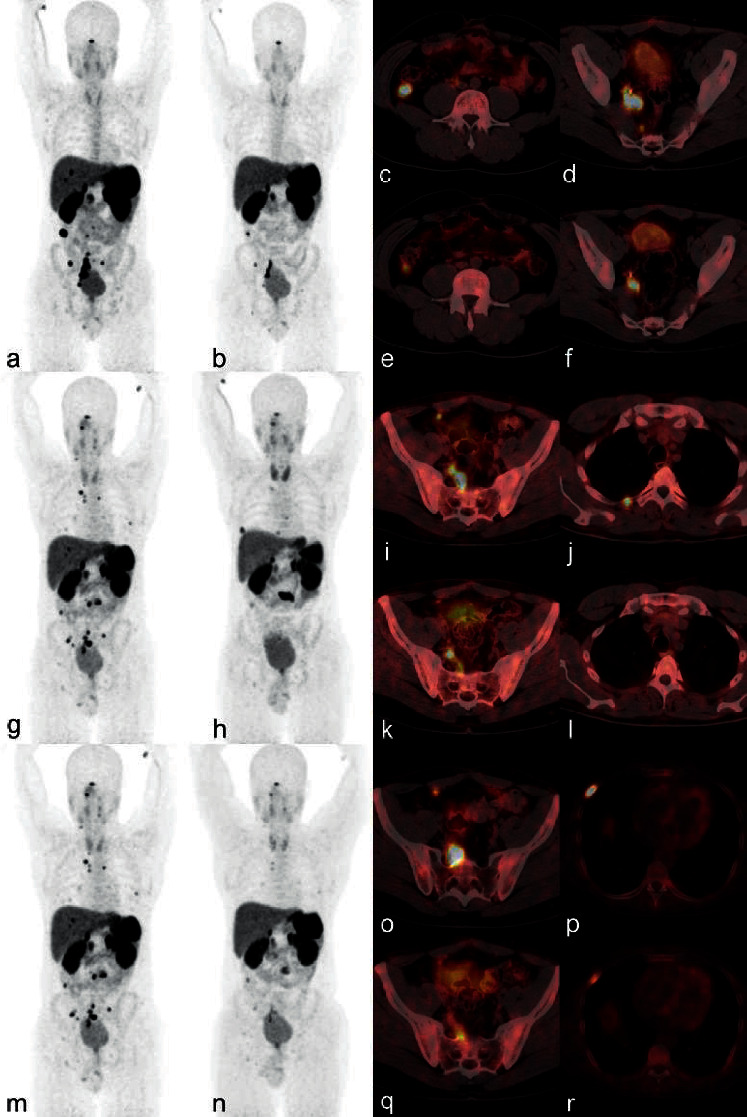
Example of the effect of the repeated tandem [^90^Y]Y/[^177^Lu]Lu-DOTATATE therapy: a 30-year-old man with a nonfunctional rectal G2 NET after surgery for primary tumours, with progression of the disease after 43 months. The [^68^Ga]Ga-DOTATATE PET/CT carried out for qualification to the PRRT showed increased uptake (higher than in the liver) in multiple, disseminated metastases. The patient received the initial PRRT, repeated PRRT, and re-repeated PRRT with the total accumulated injection activity of 29.6 GBq. The PFS rate after the I-PRRT, R-PRRT, and RR-PRRT was 35, 19, and 13 months, respectively. The disease progression after each therapy course was observed in different parts of the body. (a) [^68^Ga]Ga-DOTATATE PET MIP (maximum intensity projection) before the I-PRRT showing uptake in multiple metastases; (b) [^68^Ga]Ga-DOTATATE PET MIP after the I-PRRT showing partial response to the treatment; and axial fusion [^68^Ga]Ga-DOTATATE PET/CT (c, d) before and (e, f) after the I-PRRT. (g) [^68^Ga]Ga-DOTATATE PET MIP before the R-PRRT showing progression of the disease after the I-PRRT; (h) [^68^Ga]Ga-DOTATATE PET MIP after the R-PRRT showing partial response to the treatment; and axial fusion [^68^Ga]Ga-DOTATATE PET/CT (i, j) before and (k, l) after the R-PRRT. (m) [^68^Ga]Ga-DOTATATE PET MIP before the RR-PRRT showing progression of the disease after the R-PRRT; (n) [^68^Ga]Ga-DOTATATE PET MIP after the RR-PRRT showing partial response to the treatment; and axial fusion [^68^Ga]Ga-DOTATATE PET/CT (o, p) before and (q, r) after the RR-PRRT.

**Figure 4:**
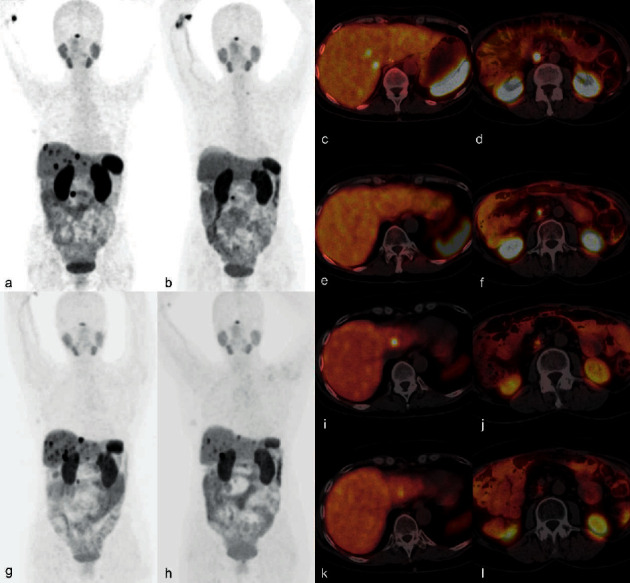
Example of the effect of the repeated tandem [^90^Y]Y/[^177^Lu]Lu-DOTATATE therapy: a 51-year-old woman with a nonfunctional pancreatic G2 NET after surgery for primary tumours, with progression of the disease after 4 months. The [^68^Ga]Ga-DOTATATE PET/CT carried out for qualification to the PRRT showed increased uptake (higher than in the liver) in multiple metastases in the liver and lymph node. The patient received the initial PRRT and the repeated PRRT with the total accumulated injection activity of 20.7 GBq. The PFS rate after the I-PRRT and the R-PRRT was 24 and 21 months, respectively. The disease progression after each therapy course was observed in the same parts of the body. (a) [^68^Ga]Ga-DOTATATE PET MIP before the I-PRRT showing increased uptake in multiple metastases; (b) [^68^Ga]Ga-DOTATATE PET MIP after the I-PRRT with complete response in the liver metastases and partial response in the lymph node; and axial fusion [^68^Ga]Ga-DOTATATE PET/CT (c, d) before and (e, f) after the I-PRRT. (g) [^68^Ga]Ga-DOTATATE PET MIP before the R-PRRT showing progression of the disease after the I-PRRT; (h) [^68^Ga]Ga-DOTATATE PET MIP after the R-PRRT with partial response; and axial fusion [^68^Ga]Ga-DOTATATE PET/CT (i, j) before and (k, l) after the R-PRRT.

At the early restaging (3–6 months) after the PRRT, PR was observed in 5 patients (19.2%) and SD in 21 patients (80.8%). After the R-PRRT, PR, SD, and PD were observed in 13 patients (50%), 11 patients (42.3%), and 2 patients (7.7%), respectively. After the RR-PRRT, PR was observed in 1 patient and SD in 4 patients (Table 4). Examples of therapeutic effects are presented in Figures 3 and 4.

## 4. Discussion

The treatment of neuroendocrine tumours is truly an interdisciplinary effort. Depending on the primary tumour location, histopathological features, and somatostatin receptor status, we have now a variety of treatment option including “cold” somatostatin analogues, mTOR inhibitors, and kinase inhibitors, as well as somatostatin-based radiotherapy. In patients with recurrence of disease, this option is also available, but cumulative side effects of a repeated therapy must always be taken into consideration.

The last 25 years of practical application of the PRRT resulted in only a few publications concerning the repeated PRRT [19–24, 29, 30], mainly with [^177^Lu]Lu-DOTATATE.

To the best of our knowledge, this is the first paper that evaluates the safety and efficacy of retreatment with simultaneous use of [^90^Y]Y- and [^177^Lu]Lu-DOTATATE. The results concern a group of 26 patients with G1 and G2 NETs.

The study published last year by van der Zwan et al. involved the highest number of patients (*n* = 181) and proved the safety and efficacy of the repeated PRRT treatment involving [^177^Lu]Lu-DOTATATE administered in a cumulative dose of up to 60.5 GBq. It was assessed that the initial and repeated PRRT have similar safety levels [23].

In our cohort, as in other publications, the majority of patients developed mild bone marrow and renal toxicity (grade 1 and 2), transient or persistent [20], which did not influence their quality of life during follow-up. MDS has developed in one patient (3.8%) who had undergone strong pretreatment with chemotherapy before the PRRT was applied. No other cases of grade 3 or 4 bone marrow toxicity were observed.

Other authors reported that 4.8–21.2% of the patients who had previously received chemotherapy developed reversible grade 3 or 4 haematological toxicity after repeated application of PRRT [19, 22, 30]. Vaughan et al. confirmed that greater bone marrow toxicity was observed in the patients treated with the PRRT using ^90^Y than with the PRRT involving ^177^Lu (41% *vs.* 28%), as well as in the patients with bone metastases [30].

There were no cases of acute myeloid leukaemia (AML) observed in the group of patients participating in this study. Van der Zwan et al. reported that the total incidence of MDS and AML among chemotherapy naive patients after the repeated PRRT was 2.2%. There were no more cases of MDS and AML occurring after the repeated PRRT than after the initial PRRT [23].

Studies concerning nephrotoxicity focus only on [^177^Lu]Lu-DOTATATE therapy without confirming grade 3 or 4 renal toxicity after repeated application of the PRRT [19, 22–24]. Grade 4 renal toxicity was observed in this study only in one patient (3.8%) with three risk factors and history of probably postinfectious acute glomerulonephritis. The remaining toxicities were all grade 1 and 2.

Therefore, our study demonstrated that up to 30.7 GBq of cumulative administered activity of the repeated PRRT with [^90^Y]Y/[^177^Lu]Lu-DOTATATE is safe, generating only a small number of side effects. This conclusion is compliant with the results of the analysis published by Sabet et al., which stated that the higher cumulative administered activity of [^177^Lu]Lu-DOTATATE (in the range of 30.0–83.7 GBq) was not associated with an increased incidence of haematotoxicity, including MDS [19].

The secondary endpoint of our study was to evaluate the benefit of progression-free survival of repeated simultaneous use of [^90^Y]Y/[^177^Lu]Lu-DOTATATE.

After the initial PRRT cycle, we observed the PFS rate of 31 months in our patients, while the PFS rate after the R-PRRT cycle lasted 23 months. Our results are in line with the conclusions of the study conducted by Severi et al. who analyzed a group with the same number of patients (26) with GEP-NET retreated with [^177^Lu]Lu-DOTATATE after an initial treatment with [^90^Y]Y-DOTATOC. According to the study's results, the post-PRRT PFS rate was 28 months, whereas the PFS rate after R-PRRT amounted to 22 months. It should be, however, noted that the cumulative administered activity was lower [20].

Van der Zwan et al. analyzed patients with progressive bronchial NET or GEP-NET who received re-retreatment with [^177^Lu]Lu-DOTATATE after benefiting from an initial application of the peptide receptor radionuclide therapy. The median PFS rate was 35.4 months after the initial PRRT, but the median PFS rate after the repeated application of the PRRT was much shorter (14.6 months). This may be explained by a bigger size of the group of patients [23]. Similar study outcomes were published in different papers. They reported that the PFS rate after the repeated PRRT cycle was shorter, ranging from 6 to 18.9 months, in comparison with the PFS rate achieved after the initial application of the PRRT [19, 22, 24, 30].

The study by Sabet et al. suggests that the PFS rate obtained after the initial PRRT may be used to predict the results of a repeated treatment [19]. Van Essen et al. reported in their study that the length of the PFS period after the initial PRRT was correlated with the PFS rate achieved after the repeated PPRT [21].

Additionally, we have evaluated the OS rate from the start of the PRRT—it amounted to 92.4 months. This OS result is favourable compared with other published data in which Vaughan et al. demonstrated the OS of 71 months [30], Yordanova et al. demonstrated the OS of 85.6 months [22], and van der Zwan et al. demonstrated the OS of 80.8 months [23].

In our study, we obtained the high disease control rate of 92.3% after the repeated PRRT. Similar DCRs of 84.6% and 84.7% were reported in the papers of Severi et al. and Rudisile et al., respectively [20, 24]. In other published publications that focused on the repeated PRRT, the reported DCR amounted to 66.6%–75% [19, 23, 30]. Differences in responses to treatment may occur due to differences in administered cumulative activities, types of radioisotope, applied response criteria, and groups of patients.

To the best of our knowledge, only Pach et al. analyzed the efficacy of the repeated tandem [^90^Y]Y/[^177^Lu]Lu-DOTATATE therapy, but on the basis of only 6 patients. The group was therefore too small for the study results to be compared with our outcomes. After 6 months, disease stabilization was observed in 5 patients and disease progression in 1 patient. The survival parameters were not assessed in the paper [25].

This study has certain limitations that ought to be taken into consideration. First of all, the study is retrospective, based on a small sample size, and includes no control group of patients. In spite of this, it provides data concerning benefits of the repeated PRRT, such as infrequent occurrences of severe toxicity, long PFS periods, and high disease control rates.

## 5. Conclusions

A repeated therapy with [^90^Y]Y/[^177^Lu]Lu-DOTATATE is safe and effective for patients with disseminated, inoperable G1 and G2 neuroendocrine tumours. The toxicity observed in the majority of patients was mild. Myelodysplastic syndrome and grade 4 renal toxicity occurred in 3.8% patients. No other cases of grade 3 or 4 bone marrow and renal toxicity were observed.

The repeated application of the PRRT results in survival benefits for the patients, and although the PFS rate is shorter than the one achieved after the initial PRRT, the effect is still reasonably good.

## Figures and Tables

**Table 1:** Patient characteristics.

Characteristic	PRRT (*n* = 26)	R-PRRT (*n* = 26)	RR-PRRT (*n* = 5)
Age (in years), mean (range)	52 (30–72)	55 (34–77)	61 (37–75)
Sex			
Male	6	6	2
Female	20	20	3
Primary tumour site			
Pancreas	11	11	2
Small intestine	5	5	—
Large intestine	8	8	2
Lung	1	1	—
Unknown	1	1	1
Grade			
1	9	9	1
2	17	17	4

**Table 2:** Bone marrow toxicity of [^90^Y]Y/[^177^Lu]Lu-DOTATATE.

*PRRT* (*n* = 26)	*n* (%)
HgB1	2 (7.7)
HgB1, transient, WBC1	1 (3.8)
HgB1, WBC1	2 (7.7)
HgB2, transient	1 (3.8)
WBC1	2 (7.7)
WBC2, transient	1 (3.8)
WBC1, PLT1, transient	2 (7.7)

*R-PRRT (n = 26)*	
HgB1	9 (34.6)
HgB1, WBC1	2 (7.7)
HgB1, WBC1, PLT1	2 (7.7)
HgB2	2 (7.7)
WBC1, transient	1 (3.8)
WBC1, PLT1	1 (3.8)
WBC2	2 (7.7)

*RR-PRRT (n = 5)*	
HgB1	3
HgB2	1
MDS	1

**Table 3:** Renal toxicity of [^90^Y]Y/[^177^Lu]Lu-DOTATATE.

*PRRT* (*n* = 26)	*n* (%)
Grade 1, transient	2 (7.7)
Grade 1	3 (11.5)
Grade 2	—
Grade 3	—
Grade 4	—

*R-PRRT (n = 26)*	*n* (%)
Grade 1, transient	1 (3.8)
Grade 1	6 (23.1)
Grade 2	2 (7.7)
Grade 3	—
Grade 4	—

*RR-PRRT (n = 5)*	*n* (%)
Grade 1	1
Grade 2	1
Grade 3	—
Grade 4	1

**Table 4:** Responses to the initial and repeated PRRT.

Group of patients	PR, *n* (%)	SD, *n* (%)	PD, *n* (%)
PRRT (*n* = 26)	5 (19.2)	21 (80.8)	—
R-PRRT (*n* = 26)	13 (50)	11 (42.3)	2 (7.7)
RR-PRRT (*n* = 5)	1	4	—

## Data Availability

The data sets analyzed during the current study are available from the corresponding author on reasonable request.
